# Towards a comprehensive and interoperable representation of consent-based data usage permissions in the German medical informatics initiative

**DOI:** 10.1186/s12911-020-01138-6

**Published:** 2020-06-05

**Authors:** Raffael Bild, Martin Bialke, Karoline Buckow, Thomas Ganslandt, Kristina Ihrig, Roland Jahns, Angela Merzweiler, Sybille Roschka, Björn Schreiweis, Sebastian Stäubert, Sven Zenker, Fabian Prasser

**Affiliations:** 1 Technical University of Munich, School of Medicine, Institute of Medical Informatics, Statistics and Epidemiology, Ismaninger Str. 22, 81675 Munich, Germany; 2 Institute for Community Medicine, Department Epidemiology of Health Care and Community Health, University Medicine Greifswald, Ellernholzstr 1-2, 17487 Greifswald, Germany; 3 TMF - Technology, Methods, and Infrastructure for Networked Medical Research, Charlottenstraße 42, 10117 Berlin, Germany; 4 Heinrich-Lanz-Center for Digital Health, University Medicine Mannheim, Heidelberg University, Theodor-Kutzer-Ufer 1-3, 68167 Mannheim, Germany; 5 Department of Medicine, Hematology/Oncology, Goethe University, Theodor-Stern-Kai 7, 60590 Frankfurt am Main, Germany; 6 Interdisciplinary Bank of Biomaterials and Data Würzburg, University and University Hospital Würzburg, Straubmühlweg 2a, 97078 Würzburg, Germany; 7 Department of Medical Information Systems, Heidelberg University Hospital, Im Neuenheimer Feld 130.3, 69120 Heidelberg, Germany; 8 Institute for Medical Informatics and Statistics, University Hospital Schleswig-Holstein and Kiel University, Arnold-Heller-Str. 3, 24105 Kiel, Germany; 9 Institute for Medical Informatics, Statistics and Epidemiology, Universität Leipzig, Härtelstraße 16-18, 04107 Leipzig, Germany; 10 Staff Unit for Medical & Scientific Technology Development & Coordination, Commercial Directorate, University Hospital Bonn, Bonn, Germany; 11 Department Of Anesthesiology & Intensive Care Medicine, University Hospital Bonn, Bonn, Germany; 12 Institute for Medical Biometrics, Informatics & Epidemiology, University of Bonn, Venusbergcampus 1, 53127 Bonn, Germany; 13 Charité - Universitätsmedizin Berlin, Charitéplatz 1, 10117 Berlin, Germany; 14 Berlin Institute of Health (BIH), Anna-Louisa-Karsch-Str. 2, 10178 Berlin, Germany

**Keywords:** Medical informatics initiative, Data integration centers, Consent template, Informed consent, Health information interoperability

## Abstract

**Background:**

The aim of the German Medical Informatics Initiative is to establish a national infrastructure for integrating and sharing health data. To this, Data Integration Centers are set up at university medical centers, which address data harmonization, information security and data protection. To capture patient consent, a common informed consent template has been developed. It consists of different modules addressing permissions for using data and biosamples. On the technical level, a common digital representation of information from signed consent templates is needed. As the partners in the initiative are free to adopt different solutions for managing consent information (e.g. IHE BPPC or HL7 FHIR Consent Resources), we had to develop an interoperability layer.

**Methods:**

First, we compiled an overview of data items required to reflect the information from the MII consent template as well as patient preferences and derived permissions. Next, we created entity-relationship diagrams to formally describe the conceptual data model underlying relevant items. We then compared this data model to conceptual models describing representations of consent information using different interoperability standards. We used the result of this comparison to derive an interoperable representation that can be mapped to common standards.

**Results:**

The digital representation needs to capture the following information: (1) version of the consent, (2) consent status for each module, and (3) period of validity of the status. We found that there is no generally accepted solution to represent status information in a manner interoperable with all relevant standards. Hence, we developed a pragmatic solution, comprising codes which describe combinations of modules with a basic set of status labels. We propose to maintain these codes in a public registry called ART-DECOR. We present concrete technical implementations of our approach using HL7 FHIR and IHE BPPC which are also compatible with the open-source consent management software gICS.

**Conclusions:**

The proposed digital representation is (1) generic enough to capture relevant information from a wide range of consent documents and data use regulations and (2) interoperable with common technical standards. We plan to extend our model to include more fine-grained status codes and rules for automated access control.

## Background

The German Medical Informatics Initiative (MII) is a large-scale, long-term strategic funding program by the German Federal Ministry of Education and Research to establish a nationwide infrastructure for the re-use and sharing of health data to improve health care and research [1, 2]. For this purpose, Data Integration Centers (DICs) are being set up at academic medical centers, which harmonize and integrate data on the local level and support processes for inter-institutional data sharing. The required organizational and technical concepts and solutions are developed jointly by four consortia, called DIFUTURE [3], HiGHmed [4], MIRACUM [5] and SMITH [6] in common Working Groups (WGs) [7] led by a National Steering Committee (NSC) consisting of representatives from all consortia.

The WG Interoperability aims to specify technical aspects of structures, processes and interfaces required to facilitate data sharing. In this context, the adoption of international standards and the involvement of corresponding expert groups are of particular importance. One import result of the group is the specification of a common National Core Dataset (NCD) [8], which defines the data structures and semantic encodings that form the technical basis of data sharing and cross-site analyses. Information security, data protection and strict adherence to patient consent with regards to the use of personal data and biosamples are further high priority topics. In this context, the WG Consent is developing a nationally harmonized template for patient information for a modular broad consent and an associated consent template (MII Informed Consent Template) [9]. This work is carried out in close cooperation with both the WG biobanking of the permanent Working Party of the German Medical Ethics Committees [10] and the WG Science of the federal data protection representatives.

On the technical level, an interoperable digital representation of information encoded in signed consent forms is needed to facilitate common data use and sharing. To develop a solution, the WGs Interoperability and Consent have formed a joint Taskforce (TF). One of the central challenges addressed by the TF was the fact that the sites participating in the MII are free to adopt different solutions for managing consent information (e.g. Integrating the Healthcare Enterprise (IHE) Basic Patient Privacy Consents (BPPC) or Health Level 7 (HL7) Fast Healthcare Interoperability Resources (FHIR) Consent Resources). Although these technologies are powerful and can represent a variety of relevant information, we found that in their entirety, they are not directly interoperable. For example, the extent to which the explicit representation of the status of a patient’s consent is covered varies. Consequently, we had to develop a harmonized common representation. An additional requirement was that the solution should not only enable the representation of status information from signed MII Informed Consent Templates but also for further consent forms and other data use policies (e.g. derogations relating to processing for scientific purposes). The TF leveraged synergies with other activities within the MII, such as the development of the NCD [8] and metadata descriptions for data sharing [11]. The developed digital representation of consent information and data use permissions is described in this article.

### Objective

An essential requirement for the digital representation presented in this article was the ability to capture relevant information from the MII Informed Consent Template, which consists of different sections addressing different use permissions. In version 1.6a, this document contains four distinct sections (use of clinical routine data, use of health insurance data, use of leftover or add-on biosamples, permission to re-contact) with a total of eight statements for which individual opt-in choices are available. We refer to such logically self-contained statements, to which individual voluntary decisions can be made, as modules [12]. In accordance with the EU General Data Protection Regulation (GDPR), active consent (“opt-in”) from patients to the statements in a specific module is required to come into effect. We call the information about whether a specific statement is *valid* or *not valid* a “*status”*. Table 1 shows an example of (possibly several) modules contained in a section about the use of clinical routine data based on the MII Informed Consent Template. We point out that all modules in this template contain the illustrated opt-in choices “Yes” and “No”, which allow patients to actively give or reject consent to the according statements.

**Table 1:** Example of a section of an informed consent template containing possibly several modules

Section		Use of clinical routine data	
**Module 1**	**Statements**	I consent to the collection, processing, storage and scientific use of my clinical routine data as described in […]	
	**Opt-in choices**	Yes	No
**Module X**	**Statements**	I consent to […]	
	**Opt-in choices**	Yes	No

In addition, there are other requirements which have been specified in the roadmap of the MII [13]. First, the digital representation must be generic so that information from other consent templates and regulations for data use can also be captured. Secondly, interoperability with regards to the technical standards used in the four consortia to manage consent information must be granted. The consortia DIFUTURE, SMITH and MIRACUM make use of the HL7 FHIR Consent Resource [14], while HiGHmed and SMITH plan to use IHE profiles such as BPPC [15] and Advanced Patient Privacy Consents (APPC) [16]. The latter will primarily be used to automatically enforce (fine-grained) access rules. We emphasize that the objective of the work described in this article was to develop an interoperable digital representation of consent information and permissions related to the use of data and/or biosamples. It was not to develop solutions for supporting the process of collecting consent information or the automated enforcement of the resulting permissions and restrictions. These issues will be addressed later in the project.

## Methods

As a first step, we compiled an overview of data items required to reflect the information from consent templates comprising several modules as well as patient preferences and derived permissions with respect to each module. Next, we created entity-relationship diagrams in order to formally describe the conceptual data model which relates these data items to each other. We then compared this data model to conceptual data models describing representations of consent information using relevant standards to analyze similarities and differences between different implementations. Based on the results of this comparison, we developed a digital representation of consent information which is interoperable with all standards used within the consortia. In this process, the TF cooperated closely with Standards Developing Organizations (SDOs), e.g. within the framework of the German Interoperability Forum [17].

## Results

### Data elements

The result of our analysis showed that in order to be able to derive permissions and restrictions regarding the use of data (and/or biosamples) on the basis of the MII Informed Consent Template, the digital representation should be able to capture at least the following data elements:

- The consent status for each module.
- The version of the consent template.
- The start date of the validity of the consent status.

The same data elements can also be used to represent information from other consents as well as policies from regulatory frameworks (e.g. research policies or law permitting certain ways of data processing without patient consent).

**Fig. 1:**
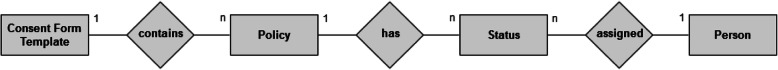
Entity-relationship diagram of the conceptional data model

Figure 1 shows the conceptual data model that captures essential entities to which these data elements can be assigned to and their relationships. It represents a minimum consensus that facilitates a common understanding and implementation across sites and enables future developments (e.g. the addition of new modules to the consent template) in a straight-forward manner. The model is deliberately kept simple and abstract in order not to anticipate details of implementation. Concrete interoperable implementation options with standards and tools used by the participating sites will be described in subsequent sections.

### General concept

An important result of our cooperation with the SDOs was that at the time being, there are no generally accepted code systems available with values that can serve as a basis for an interoperable digital representation of the status of consents or policies. In addition, relevant standards such as HL7 FHIR are still under development. For example, the status codes of the FHIR Consent Resource [14] are not yet finalized. The IHE BPPC profile, on the other hand, does not support the explicit inclusion of status codes [15]. To overcome these limitations in the future, the MII is actively engaged in the development of the solutions mentioned, for example in the context of the WG on Consent Management of the Interoperability Forum [17, 18].

To provide a solution that ensures interoperability across sites, we have developed the pragmatic approach described in this article. Three status labels are used: “valid”, “not valid” and “unknown”. These labels indicate whether or not the permissions or restrictions resulting from the referenced module of a consent template or other regulatory policies (henceforth, we refer to consent modules or other policies on data use simply as *policies*) are in effect. This information is sufficient to represent patient consent and legal frameworks in an interoperable manner. Moreover, status information supported by the standards and consent management solutions used within the consortia can be mapped to these labels (see Section 4).

On the implementation level, we defined a *code system* to distinguish whether data use policies are in effect or not. In this context, *code system* denotes a set of codes with well-defined identifiers and well-defined semantics. Each code in the proposed code system identifies – due to the mentioned restrictions of relevant standards – a combination of a specific policy with one of the three status labels introduced above. Code systems can be referenced by Object Identifiers (OIDs) [19] which denote a node in a hierarchically structured namespace having the structure of a tree. An OID takes the form of a series of integers separated by dots which corresponds to the path from the root node to the node in question. An OID, in turn, can be represented as a Uniform Resource Name (URN) [20], a common kind of internet resource identifier. Our proposed code system can be referenced by the OID 2.16.840.1.113883.3.1937. 777.24.5.1 or the corresponding URN urn:oid:2.16.840.1.113883.3.1937.777.24.5.1. Each code within the system can be referenced using an OID, satisfying the requirements of BPPC [21], or using an URN, satisfying the requirements of the HL7 FHIR Consent Resource [14].

The TF Core Dataset of the MII is currently using ART-DECOR to develop the MII Core Dataset. ART-DECOR is an open-source software for the creation of HL7 templates, value sets, scenarios and datasets [22]. Consequently, we decided to manage the code systems for the MII Informed Consent Template through ART-DECOR as well [23]. In these code systems the codes are defined in such a way that they clearly represent policies in a specific version of the consent template. Common *value sets* combine all codes that refer to a specific policy of the consent template. A value set is a selection of codes that may have been defined in different code systems and they specify which codes can be used in which context [24]. Each value set in the context of the MII Informed Consent Template is linked with a description which also contains the version number of the consent template.

**Fig. 2:**
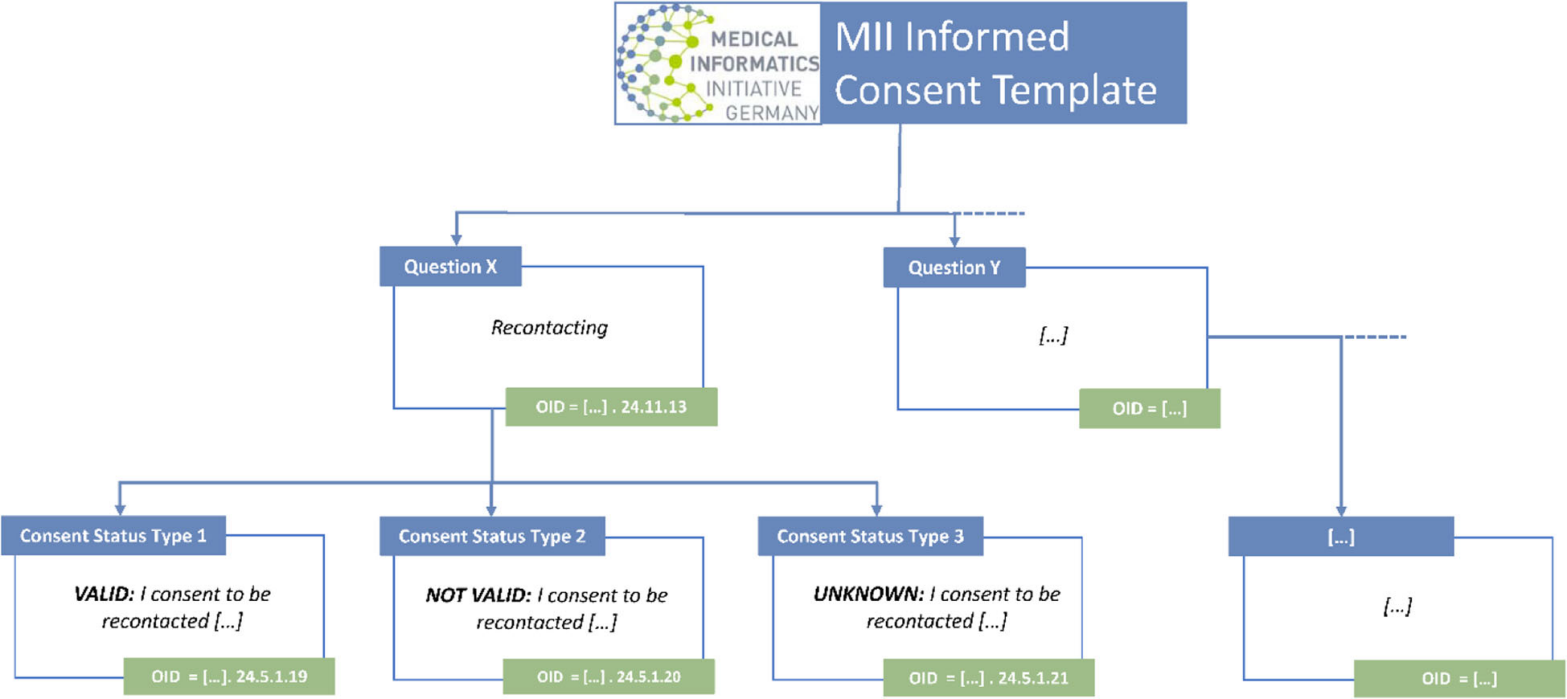
Example representation of information captured through the MII Informed Consent template: Using ART- DECOR, each question and related combination of answer-text and consent status type is assigned a unique consecutive object identifier (OID)

Figure 2 shows an example illustrating how information captured through the MII Informed Consent Template is represented in ART-DECOR. As can be seen, for each question and each associated combination of answer-text and consent status type, a unique consecutive object identifier has been defined. As an example, the figure shows the OIDs associated with the module asking for consent to be re-contacted for purposes such as information about further research projects. For other modules, including further modules dealing with consent to be re-contacted for other reasons, different OIDs have been defined analogously.

In addition to the corresponding policies and status information, the initial date of the validity of the status (not to be confused with the validity of the policy referenced by the status label) must also be documented. Optionally, the end of the period of validity can also be documented. For example, the MII Informed Consent Template contains modules asking for consent to the use of health insurance data for a maximum of five years. Obviously, it is also necessary that the patient to whom a digitally represented status refers is referenced. This is supported by all implementations described in the next section. If consent to a policy is withdrawn, this can be documented by creating a corresponding instance of the digital documentation referencing the code representing the status label “not valid” for this policy.

As mentioned above, the proposed digital representation can not only be used to represent information from consent templates, but also from other contexts, such as regulatory policies. For example, the Bavarian Hospital Act [25] states that hospital physicians are allowed to use clinical routine data for intramural research. Similar to statements from consent templates, such statements from regulatory policies can also be combined with status codes, and those combinations can then be referenced via unique identifiers. This can act as a documentation for whether a specific policy is valid in a specific context (e.g. for data from a specific hospital).

### Implementation with HL7 FHIR and IHE BPPC

In HL7 FHIR, status information for policies can be implemented with the Consent resource [14]. The codes from the code system described in Section 3.2 can be referenced using the attribute “policy”. The date of the beginning (and if applicable the end) of the validity of the resource itself can be represented by the attribute “period”. The person the resource refers to can be referenced via the “patient” attribute. In this way, the content described in the previous section can be represented both with the current FHIR Release 4 (v4.0.0) and with earlier versions, such as Release 3 (v3.0.1). Table 2 shows an example of a FHIR Release 4 Consent resource.

**Table 2:** Example representation using a HL7 FHIR Consent resource

Code	Description
{ “resourceType”: “Consent”, “status”: “active”,	Type definition
“scope”: { “coding”: [ { “system”: “http://terminology.hl7.org/CodeSystem/consentscope”, “code”: “patient-privacy” } ] },	States that the represented information refers to “Agreement to collect, access, use or disclose (share) information”
“category”: [ { “coding”: [ { “system”: “http://loinc.org”, “code”: “57,016–8” } ] } ],	States that the resource is based on a consent document
“patient”: { “identifier”: { “value”: “patientf001” } },	References the person
“policy”: [ { “uri”: “http://art-decor.org/decor/services/RetrieveCode?code=2.16.840.1.113883.3.1937.777.24.5.1.1&codeSystem=2.16.840.1.113883.3.1937.777.24.5.1” } ],	List of codes
“provision”: { “period”: { “start”: “2018-10-10”, “end”: “2023-10-09” } }	Period of validity
}	

IHE BPPC supports the digital documentation of consent information in the form of CDA R2 documents [15, 26]. The contents presented in this article can be represented in the CDA header of the documents by using the XML element “serviceEvent”. This element can represent the codes of the specified code systems via the XML element “code” and information about the validity period via the XML element “effectiveTime”. The patient or proband to which the encoded information refers to is specified in the XML element “patientRole”. Table 3 illustrates an example of implementation with IHE BPPC. It shows an interoperable representation of the information also shown in Table 2.

**Table 3:** Example representation using BPPC

Code	Description
<ClinicalDocument xmlns = ‘urn:hl7-org:v3’> <templateId root = ‘1.3.6.1.4.1.19376.1.5.3.1.1.1’/> <templateId root = ‘1.3.6.1.4.1.19376.1.5.3.1.1.7’/> < code code = ‘57,016–8’ displayName = ‘PATIENT PRIVACY ACKNOWLEDGEMENT’ codeSystem = ‘2.16.840.1.113883.6.1’ codeSystemName = ‘LOINC’/>	Type definition
<recordTarget> <patientRole> <id extension = “0411886319605719371016” root = “1.3.6.1.4.1.19376.1.8.9.2”/> <addr use = “HP”> ... </addr> <patient> ... </patient> </patientRole> </recordTarget>	Information about the person
<documentationOf typeCode = ‘DOC’> <serviceEvent classCode = ‘ACT’ moodCode = ‘EVN’> <templateId root = ‘1.3.6.1.4.1.19376.1.5.3.1.2.6’/> <id root = ‘1.2.3.4.5.6’/>	
<code code = ‘2.16.840.1.113883.3.1937.777.24.5.1.1’ codeSystem = ‘2.16.840.1.113883.3.1937.777.24.5.1/>	List of codes
<effectiveTime> <low value = ‘20181010’/> <high value = ‘20231009’/> </effectiveTime>	Period of validity
</serviceEvent> </documentationOf> <component> <structuredBody> ... </structuredBody> </component> </ClinicalDocument>	

## Discussion

The primary aim of the digital representation described in this article is to provide a cross-site interoperability layer for representing the validity of data use policies derived from signed informed consent templates and regulatory frameworks. The technical implementations described can be mapped to each other without loss of information, since we created code systems to achieve semantic interoperability. In addition, compatibility is provided with more complex implementations utilized by the MII partner sites. If, for example, HL7 FHIR is used to support the process of consent management, all status codes provided in Release 3 (v3.0.1) and Release 4 (v4.0.0) can be mapped to the status codes mentioned in Section 3.2. Table 4 shows such a mapping.

**Table 4:** Mapping of status labels from the FHIR Consent resource to the proposed digital representation

Label in FHIR	“draft”	“proposed”	“active”	“rejected”	“inactive”	“entered-in-error”
**Mapping**	“not valid”	“not valid”	“valid”	“not valid”	“not valid”	“not valid”

The same applies to the status codes used by the open source software gICS [27, 28], which was developed by the University Medicine Greifswald, Germany and is part of the technical tools used by the MIRACUM consortium to manage informed consents and/or withdrawals. gICS supports the documentation of the required data items and allows to define the necessary validity period of consents. All four consortia plan to use gICS to manage the respective consent templates and to map OIDs to the original textual representation. Moreover, gICS facilitates the capability to execute OID-specific queries for consent states of MII-patients (e.g. OID-specific queries relating to a specific question or answer for all respective consents or patients who consented). Table 5 shows a mapping of the status labels used by gICS (Version 2.8.6) to the labels used by the interoperable representation developed.

**Table 5:** Mapping status labels from gICS to the proposed digital representation

Label in gICS	“Accepted”	“Declined”	“Withdrawn”	“Invalidated”
**Mapping**	“valid”	“not valid”	“not valid”	“not valid”

The use of MII consent status codes (valid, not valid, unknown) enables the establishment of an interoperability layer across all consortia. However, due to the minimalistic approach of the common representation, the mapping of data from more complex implementations (e.g. withdrawn, invalidated, pending, etc.) into the interoperability layer is associated with loss of information. Our representation preserves all data that is needed to decide upon common data use, but it cannot serve as a basis for implementing management processes.

In the National Core Dataset defined by the MII, individual data elements are also grouped into so-called modules (not to be confused with the modules of a consent template). Future developments of the dataset include the “consent” module, which is to include information on patient informed consent [8]. The digital representation described in this article lays important groundwork for the development of this module. Further synergies within the MII exist with the development of common metadata descriptions on data availability and use [9], which are developed in addition to the National Core Dataset.

In future work, we plan to extend the digital representation in such a way that also rules for automated access control can be included. For example, the HL7 FHIR Consent resource supports the representation of „policyRules “and IHE APPC documents representing such rules can be derived from BPPC [29]. These extensions be based on additional standards, such as XACML [30] or ADA-M [31], and they will make use of additional information captured by our solution, e.g. on consent validity periods (see Section 3.2). Moreover, we plan to support additional status codes to further facilitate harmonization of consent management processes, e.g. by explicitly representing states such as “withdrawn“ or “decision pending“ instead of summarizing them under a common state “not valid“. This will take more time, however, as it will also require changes to the standards and tools currently used by the MII sites. Finally, the withdrawal of consent “*shall be as easy [...] as to give consent*” (EU GDPR [32], ch. 2, art. 7, lit. 4). This suggests that a common MII template for withdrawals needs to be implemented as well and integrated into the technical framework presented in this article.

## Conclusions

We have presented a digital representation of information from signed consent templates developed in the German MII. Our solution is generic, as it supports representing information from signed instances of the MII Informed Consent Template as well as other consent templates and policies from data use regulations. It is compatible with the technical standards used at the participating sites, in particular to HL7 FHIR in versions 3 and 4 as well as IHE BPPC, and hence forms a cross-site interoperability layer for data use policies. Our results also provide an important basis for further developments within the MII, including extensions of the National Core Dataset and the development of automated access control processes.

The proposed solution for an interoperable representation of the information on the consent status is based on individual codes per distinct combination of a specified policy and its validity. If the wording of text passages is changed for legal or ethical reasons or new modules are added, a modification or extension of the respective ART-DECOR OIDs will be required. The coordination of such modifications will require additional organizational efforts, which will be implemented by utilizing the governance framework already set up within the MII.

## Data Availability

The code systems and value sets developed for representing the MII Informed Consent are publicly available on ART-DECOR [23].

## Abbreviations

MII: Medical Informatics Initiative
DIC: Data Integration Center
WG: Working Group
NSC: National Steering Committee
NCD: National Core Dataset
TF: Taskforce
IHE: Integrating the Healthcare Enterprise
BPPC: Basic Patient Privacy Consents
HL7: Health Level 7
FHIR: Fast Healthcare Interoperability Resources
GDPR: General Data Protection Regulation
APPC: Advanced Patient Privacy Consents
SDO: Standardization organization
OID: Object Identifier
URN: Uniform Resource Name
